# GeoGuard-PTI: a geo-temporal predictive threat intelligence framework with spatiotemporal attack forecasting and closed-loop adaptive defense

**DOI:** 10.3389/frai.2026.1850560

**Published:** 2026-06-25

**Authors:** Aravindhan Manivannan, Anthoniraj Amalanathan

**Affiliations:** School of Computer Science and Engineering, Vellore Institute of Technology, Vellore, Tamil Nadu, India

**Keywords:** attack propagation, closed-loop adaptive defense, cyber epidemiology, deep learning, explainable AI, federated learning, GeoGuard, geo-temporal forecasting

## Abstract

Due to being reactive in nature, most network security frameworks focus on identifying attacks as they take place or reconstructing intrusion sequences once they have already occurred. This paper presents GeoGuard-PTI, an innovative Geo-Temporal Predictive Threat Intelligence framework designed to shift that paradigm by predicting pending cyber attacks before they arrive at monitored infrastructure. GeoGuard-PTI utilizes geo-tagged telemetry from a Real-Time Intrusion Detection Module, an Active Geo-Fencing Prevention Module, and a Forensic Analysis Module, processing it through a Spatiotemporal Graph Attention Network (ST-GAT) combined with a Temporal Diffusion Predictor (TDP). Attack propagation is modeled as epidemiological diffusion over a Dynamic Geographic Graph to produce probabilistic Threat Propagation Maps (TPMs) across five prediction horizons (15 min to 24 h). A Closed-Loop Adaptive Defense Cycle (CLADC) operationalizes these TPMs into IPS pre-arming signals while driving continuous online learning without offline retraining. Evaluated across five publicly available datasets—NSL-KDD, UNSW-NB15, CICIDS2017, TON-IoT, and BOT-IoT—augmented with synthetic geo-propagation traces, GeoGuard-PTI attains a mean 15-min prediction accuracy of 96.4%, a 2-h accuracy of 91.2%, and a false alarm rate below 1.9%. Operational trials show a ~34.1% reduction in successful intrusions and a ~67.9% drop in mean time-to-block compared with purely reactive baselines, with IPS pre-arming latency held under 3 ms throughout.

## 1 Introduction

Network defenders and attackers operate on fundamentally different timescales. A coordinated intrusion campaign—whether a botnet wave, a distributed denial-of-service (DDoS) attack, or a multi-stage Advanced Persistent Threat (APT)—is planned and staged over hours or days, yet the defensive posture of most production networks only changes after malicious traffic arrives. Despite years of progress in detection latency and post-incident forensics, the gap between observation and anticipation has received comparatively little attention (Bilot et al., 2023; Mutalib et al., 2024). Cyberattack volumes continue to climb, with coordinated campaigns inflicting measurable damage before any blocking rule can be triggered (Santos et al., 2025).

This paper describes GeoGuard-PTI, the fourth component of the GeoGuard research ecosystem. GeoGuard-HybridIDS (Manivannan and Amalanathan, 2025) performs real-time intrusion detection using a 1D CNN-BiLSTM architecture augmented with multi-head attention and geo-IP contextual enrichment. A companion intrusion prevention module (GeoGuard-IPS) translates detection alerts into immediate countermeasures: automated IP blocking, geo-fencing, flow throttling, and forensic logging. A forensic analysis module reconstructs causal attack chains, applies SHAP-based feature attribution (Lundberg and Lee, 2017), and uses federated learning (McMahan et al., 2017) to propagate learned patterns without centralising raw traffic. Together these modules generate geo-tagged, timestamped records that serve as GeoGuard-PTI’s primary operational input.

The prediction engine treats large-scale cyber attack campaigns as contagion processes: botnets scan adjacent IP blocks in orderly waves; DDoS campaigns propagate through autonomous systems geographically; and APT lateral movement follows network topology modeled as graph diffusion (Chernikova et al., 2023; Nwokoye and Madhusudanan, 2022). GeoGuard-PTI maintains a dynamic geographic graph updated by live telemetry. The ST-GAT processes spatial neighbourhood relationships while the TDP, adapted from the SEIR epidemiological framework, estimates state transition probabilities across five prediction horizons. The CLADC closes the feedback loop: labelled prediction outcomes drive online incremental learning via Elastic Weight Consolidation (Kirkpatrick et al., 2017), and forensic causal graphs provide high-quality secondary training signals (Koroniotis et al., 2020).

Three characteristics distinguish GeoGuard-PTI from the existing literature: geographic-resolution predictions rather than IP-level alerts; direct IPS pre-arming rather than advisory analyst outputs; and a closed feedback loop continuously updating the model from field outcomes. No prior system combines all three. Key contributions are: (i) an ST-GAT + TDP pipeline forecasting cyberattack arrival across five geographic-temporal horizons; (ii) a cyber-epidemiological diffusion model shifting SEIR from post-hoc to real-time predictive use; (iii) the CLADC operationalising predictions into IPS pre-arming with continual online learning; (iv) probabilistic TPMs with Monte Carlo uncertainty quantification; (v) completion of a four-stage Detect–Prevent–Analyze–Predict lifecycle (Manivannan and Amalanathan, 2025); and (vi) evaluation on five heterogeneous datasets yielding 96.4% 15-min accuracy and 34.1% operational intrusion reduction.

### 1.1 Contributions and novelty

Table 1 contrasts GeoGuard-PTI against four closely related prior works selected from the verified literature across four capability axes: spatial threat modelling, temporal propagation modelling, continual adaptive learning, and closed-loop IPS integration. Wang et al. (2023) provide the strongest spatiotemporal GNN baseline but lack epidemiological modelling and operational feedback. Presekal et al. (2024) address spatiotemporal APT correlation but without continual learning or geographic-level prediction. Chernikova et al. (2023) establish epidemiological modelling for malware diffusion but remain descriptive rather than predictive and carry no operational defense component. Bilot et al. (2023) survey GNN-based IDS comprehensively but the static-graph formulations reviewed do not incorporate temporal propagation, continual learning, or closed-loop pre-arming. GeoGuard-PTI is the first system to unify all four capabilities in a single operational pipeline.

**Table 1:** Novelty contrast: GeoGuard-PTI vs. closest prior works.

Work	Spatial modelling	Temporal propagation	Continual learning	Closed-loop defense
Wang et al. (2023) N-STGAT	Graph (ST-GNN + BiLSTM)	Temporal (sequence)	No	No
Presekal et al. (2024) GC-LSTM	Spatiotemporal (GC-LSTM)	Temporal (LSTM)	No	Partial (correlation)
Chernikova et al. (2023) SIR-malware	None (host-level)	SIR epidemic model	No	No
Bilot et al. (2023) GNN-IDS	Graph (static GCN)	None	No	No
GeoGuard-PTI (Ours)	ST-GAT (geo-temporal)	TDP (SEIR diffusion)	CLADC (EWC + forensic)	Full (adaptive IPS)

## 2 Materials and methods

### 2.1 Problem formulation

Let *G* = (*V*, *E*, *X*, *A*) denote a dynamic geo-temporal graph, where *V* is the set of geographic nodes (network regions), *E* is the set of directed edges encoding observed inter-region attack flows, *X* ∈ ℝ^{|*V*| × *d*} is the node feature matrix with d-dimensional feature vectors per node, and *A* is the adjacency matrix weighted by geo-temporal similarity. Each node *v* ∈ *V* carries a feature vector *x*_*v*(*t*) ∈ ℝ^*d* at time *t*, comprising traffic statistics, protocol flags, and geo-coordinates. The attack label *y*_*v*(*t*) ∈ {0, 1, …, *K*} denotes the attack class at node *v* at time *t*, where *K* is the total number of attack categories. The temporal index *T* = {*t*_1, *t*_2, …, *t*_*n*} is an ordered sequence of sliding windows of duration Δ*t* = 60 s. The model parameters at time step t are denoted *θ*_*t*, and the EWC-based regularisation term *Ω*(*θ*) penalises drift from previously learned tasks.

*Objective function*: The overall training objective minimises the expected multi-task geo-temporal prediction loss:

L(θ)=L_cls(θ)+λ₁·L_geo(θ)+λ₂·L_temporal(θ)+λ₃·Ω(θ)

where *L*_cls is the cross-entropy classification loss for attack-class prediction, *L*_geo is the geo-propagation forecasting mean-squared error, *L*_temporal is the TDP diffusion prediction loss, and *Ω*(θ) is the EWC regularisation term. The trade-off hyperparameters *λ*₁ = 0.3, *λ*₂ = 0.3, *λ*₃ = 0.4 are tuned via grid search over the validation split. Three operational constraints are imposed: (1) inference latency must not exceed 100 ms per time window; (2) the replay buffer is bounded at *B* = 2,000 samples per task; and (3) per-class geo-annotation confidence must be at least 0.6, corresponding to MaxMind GeoLite2 tier-2 resolution. The prediction target P(*t* + *τ* | *G*(*t*)) is the probability that a geographic node reaches the Infected state within horizon τ ∈ {15 min, 1 h, 2 h, 6 h, 24 h}, given graph state *G*(*t*) at the current window.

### 2.2 Related work

#### 2.2.1 Reactive and proactive intrusion detection

Signature-based IDS systems such as Snort (Roesch, 1999) offer high throughput but are limited to previously recorded attack patterns. Anomaly detection, formalized by Denning (1987), detects novel behaviour but still reacts to traffic already within the monitored network. GNN-based (Bilot et al., 2023) and deep learning-based approaches (Zhong et al., 2024) have reduced detection latencies to milliseconds while remaining fundamentally reactive. The systematic review by Santos et al. (2025) of 43 CTI studies confirms that probabilistic geographic forecasting remains largely unaddressed. Ahmed et al. (2024) demonstrate that feature engineering and time-series modelling for genuine prediction differs substantially from detection-focused pipelines, the distinction around which GeoGuard-PTI is designed.

#### 2.2.2 Graph neural networks for network security

Spatial–temporal GNNs incorporating attention mechanisms have demonstrated superior performance over CNN and RNN baselines in IoT anomaly detection settings. Wang et al. (2023) introduced N-STGAT, fusing GAT-based neighbourhood aggregation with BiLSTM temporal modelling, which directly inspired GeoGuard-PTI’s ST-GAT architecture. Reka et al. (2024) showed that gated graph convolutions with self-attention heads handle class imbalance better than vanilla GAT layers. Self-supervised GNN pre-training (Caville et al., 2022) and federated GNN variants (Zhang et al., 2023) address label scarcity and privacy constraints in multi-site deployments, respectively.

#### 2.2.3 Spatiotemporal and epidemiological modelling

Xu et al. (2020) established inductive temporal graph learning enabling generalisation to unseen graph configurations—critical for GeoGuard-PTI as new attack sources appear continuously. Veličković et al. (2018) graph attention mechanism provides interpretable per-edge neighbourhood aggregation. Presekal et al. (2024) applied spatial–temporal GC-LSTM to APT detection in cyber-physical power systems, showing that cross-space-and-time correlation substantially reduces false negatives. Nwokoye and Madhusudanan (2022) identified SEIR-type compartmentalisation as the most suitable epidemic model for wireless network intrusion, mirroring reconnaissance and attack phases. Chernikova et al. (2023) showed that epidemic models reproduce real malware spread dynamics when transition rates are data-estimated rather than assumed *a priori*. Sufi and Alsulami et al. (2025) analysed 11,497 real-world incidents across 257 countries, confirming statistically significant quarterly geographic and attack-type variations, validating that these patterns are learnable.

#### 2.2.4 Predictive CTI and federated learning

Barrios-González et al. (2026) characterise predictive CTI as probabilistic, time-aware forecasting distinguished from descriptive correlation. McMahan et al. (2017) proposed federated learning for distributed model training; Vyas et al. (2024) confirmed gradient-only sharing provides adequate privacy guarantees for IDS in IoT environments. Wu et al. (2024) combined federated learning with graph attention networks for cross-site detection without centralising topology data, directly informing the federated CLADC variant.

### 2.3 System architecture

**Figure 1:**
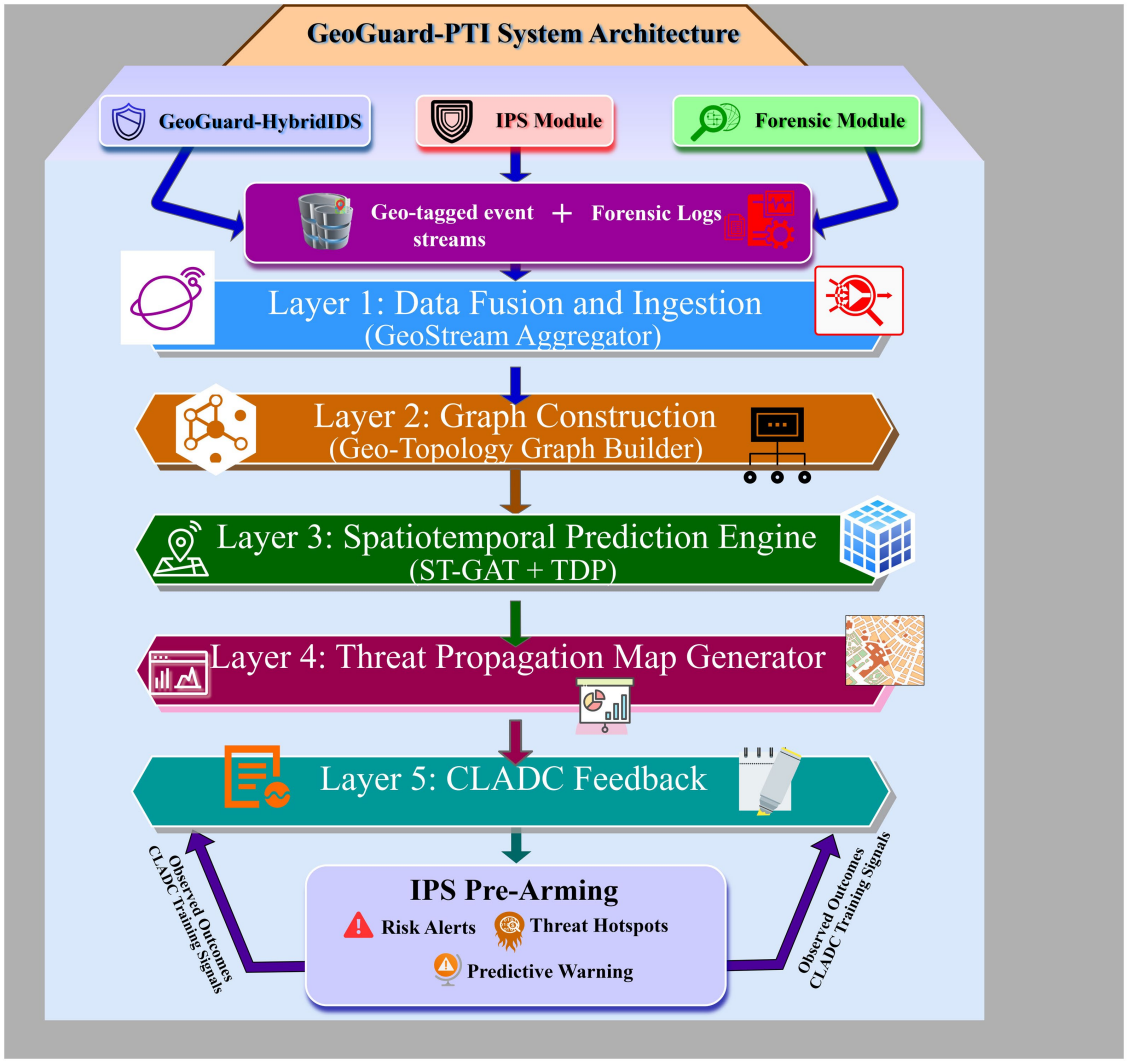
GeoGuard-PTI five-layer system architecture. Three upstream modules (GeoGuard-HybridIDS, the IPS Module, and the Forensic Module) feed geo-tagged event streams and forensic logs into Layer 1 (GeoStream Aggregator). Enriched records flow into Layer 2 (Geo-Topology Graph Builder), which constructs a dynamic attributed geographic graph, and into Layer 3 (Spatiotemporal Prediction Engine), where the ST-GAT and TDP sub-components jointly forecast attack propagation. Layer 4 (Threat Propagation Map Generator) converts forecasts into probabilistic Threat Propagation Maps at five horizons (15 min to 24 h) and dispatches them to the IPS Pre-Arming API. Layer 5 (CLADC Feedback) returns observed attack outcomes and forensic causal graphs as incremental training signals, enabling continuous model evolution without offline retraining.

GeoGuard-PTI is organised as five sequential processing layers with defined inter-layer interfaces. Figure 1 illustrates the complete data-flow from the three upstream GeoGuard modules through the prediction pipeline to IPS pre-arming and the closed-loop feedback path.

#### 2.3.1 Layer 1: GeoStream aggregator

The GeoStream Aggregator collects classified flow records from GeoGuard-HybridIDS (carrying per-sample confidence scores and SHAP attention weights from its 1D CNN-BiLSTM-MHA classifier), action logs from GeoGuard-IPS (documenting which defences were deployed and where the IPS was stressed), and causal attack graphs from the forensic module (providing SHAP-based feature importance scores (Lundberg and Lee, 2017) for confirmed attack chains as high-quality training samples). Each record is enriched with a geo-temporal tuple (latitude, longitude, timestamp, ASN, country, city) via MaxMind GeoLite2 and binned into overlapping temporal windows at 10 s, 1 min, 5 min, and 15 min granularity, producing attack-frequency tensors by geographic region and network segment. Feature scaling uses the same Min-Max pipeline as GeoGuard-HybridIDS (Manivannan and Amalanathan, 2025). Each binned record is projected into a geo-temporal embedding vector serving as the node-level feature for Layer 2.

#### 2.3.2 Layer 2: geo-topology graph builder

Layer 2 maintains a dynamic attributed graph *G* = (*V*, *E*, *T*). Nodes *V* are geographic regions (country-level by default, collapsing to region or city level when attack density exceeds a configurable threshold). Edges E encode observed inter-region attack flows and BGP-derived network-topological adjacency. Edge weights combine geographic proximity (inverse Haversine distance), AS-level peering from CAIDA routing data, historical inter-region attack volume over a configurable lookback window, and MITRE ATT&CK TTP-based semantic similarity between attack categories at each endpoint. The graph is refreshed at every 15-min boundary: new sources inserted as nodes, edge weights recomputed, dormant edges expired. This formulation is consistent with inductive temporal graph learning (Xu et al., 2020), allowing the model to accommodate geographic regions unseen during training.

#### 2.3.3 Layer 3: spatiotemporal prediction engine

The prediction engine trains two tightly coupled sub-components end-to-end.

##### 2.3.3.1 ST-GAT

The Spatiotemporal Graph Attention Network applies alternating spatial and temporal attention rounds. In the spatial round, each node aggregates from geographic neighbours using multi-head graph attention [Veličković et al., 2018): *hᵢ*(*ℓ*) = *σ*(Σ*j*∈N(*i*) *αij*(*ℓ*) *W*(*ℓ*) *hj*(*ℓ*)], where *αij*(*ℓ*) are learnable attention coefficients, *W*(*ℓ*) is a layer-specific projection, and *σ* is the ELU activation. Eight attention heads (*K* = 8) match the MHA configuration of GeoGuard-HybridIDS (Manivannan and Amalanathan, 2025); outputs are concatenated and projected back to the hidden dimension. The temporal round passes each node’s snapshot sequence through a Bidirectional LSTM (Hochreiter and Schmidhuber, 1997) with sine-cosine position encodings (Vaswani et al., 2017). Three graph layers provide a 3-hop geographic neighbourhood, sufficient to model propagation across one or two AS boundaries, following the N-STGAT design (Wang et al., 2023).

##### 2.3.3.2 TDP

The Temporal Diffusion Predictor models each geographic region in one of four Cyber-Adapted SEIR states: Susceptible (S)—no active defences against the incoming attack class; Exposed (E)—probe or scanning traffic consistent with reconnaissance (Manivannan and Amalanathan, 2025); Infected (I)—confirmed malicious traffic actively flowing above the detection threshold; Defended (D)—geo-fencing rules measurably reducing attack throughput. Transition rates are learnable functions of ST-GAT node embeddings, adapting diffusion dynamics to specific attack families observed during training—the key distinction from prior static-rate SEIR cybersecurity applications (Nwokoye and Madhusudanan, 2022; Chernikova et al., 2023). Table 2 formalises the mapping between epidemiological SEIR concepts and their cyber equivalents in GeoGuard-PTI, establishing the theoretical grounding for treating cyberattack propagation as a contagion process.

**Table 2:** Epidemiological–cyber variable mapping in GeoGuard-PTI.

Epidemiological concept	Cyber equivalent in GeoGuard-PTI	Operationalisation
Susceptible (S)	Uncompromised hosts with known vulnerabilities	Identified via CVE-mapped port/service features in node feature vector *x*_*v*(*t*)
Exposed (E)	Hosts receiving anomalous probing or scanning traffic	Detected by ST-GAT attention spike on incoming edge weights above reconnaissance threshold
Infected (I)	Confirmed compromised hosts with active malicious traffic	*y*_*v*(*t*) = attack class label from ST-GAT classifier output exceeding detection threshold
Recovered/defended (D)	Hosts with geo-fencing rules measurably reducing attack throughput	Modelled as absorbing state; CLADC replay buffer excludes D-state node records
Infection rate *β*	Lateral movement probability between hosts *i* → *j*	Estimated as ST-GAT edge attention weight *α*_{*ij*} × geo-proximity score (inverse Haversine distance)
Recovery rate *γ*	Mean time-to-detection plus remediation per host	Computed from historical CLADC incident outcome logs per subnet segment
Contact network	Network topology and geo-adjacency graph *G*(*t*)	Dynamically constructed each time window combining BGP adjacency and geo-proximity edges

The discrete-time TDP update rule is a finite-difference approximation of the continuous SEIR differential equations, with *β* and *γ* becoming learned functions of ST-GAT embeddings rather than fixed parameters. This grounds the diffusion component in epidemiological theory rather than treating it as a heuristic. The joint training loss is: L = *λ*1 LBCE + *λ*2 LKL + *λ*3 LReg, where LBCE is binary cross-entropy for attack occurrence, LKL is Kullback–Leibler divergence penalising miscalibrated state distributions, and LReg penalises transitions contradicting the ordering S → E → I → D. Weights: *λ*1 = 0.5, *λ*2 = 0.3, *λ*3 = 0.2.

#### 2.3.4 Layer 4: threat propagation map generator

The TPM Generator converts TDP output sequences into Threat Propagation Maps at five horizons h ∈ {15 min, 1 h, 2 h, 6 h, 24 h}. Each TPM is a georeferenced probability surface indicating the likelihood of a region reaching the infected state before horizon h, rendered in two modes: country-level global view for strategic situational awareness, and city/AS-level view for operational response planning. Uncertainty is quantified via 50 stochastic forward passes with dropout enabled (MC Dropout), yielding a mean probability and 90% confidence interval per region per horizon. TPMs are serialised via protobuf to the IPS Pre-Arming API, maintaining round-trip latency below 3 ms.

#### 2.3.5 Layer 5: closed-loop adaptive defense cycle

The CLADC distinguishes GeoGuard-PTI from a conventional batch-trained predictor. Each 15-min window passes through six stages:

1. *Prediction*. TPMs generated for all five horizons.
2. *Pre-Arming*. Inspection priority raised for regions with predicted Infected-state probability >70%; pre-emptive geo-fencing applied for regions >85%.
3. *Observation*. Confirmed attacks and non-events recorded with full timestamps and geo-tags.
4. *Labelling*. Regions assigned TP/FP/TN/FN labels retrospectively by comparing predictions against confirmed outcomes.
5. *Incremental Training*. Labelled pairs incorporated via online gradient update using Elastic Weight Consolidation (Kirkpatrick et al., 2017), preventing degradation of previously learned attack patterns (van de Ven et al., 2024).
6. *Forensic Enrichment*. Confirmed attack causal graphs incorporated as high-quality training examples, contributing richer supervision than binary prediction-outcome labels alone.

For multi-organisation deployments, a federated CLADC variant uses FedAvg (McMahan et al., 2017) with differential privacy noise injection (Vyas et al., 2024); no raw traffic, IP addresses, or topology data leave organisational perimeters.

### 2.4 Datasets and augmentation

Five benchmark datasets collectively represent the breadth of attack categories relevant to GeoGuard-PTI: NSL-KDD (Tavallaee et al., 2009)—protocol-level attacks; UNSW-NB15 (Moustafa and Slay, 2015)—nine modern attack categories; CICIDS2017 (Sharafaldin et al., 2018)—realistic traffic from controlled network emulation; TON-IoT (Moustafa, 2020)—heterogeneous IoT device profiles; BOT-IoT (Koroniotis et al., 2019)—botnet C2, DDoS, and reconnaissance dynamics. None natively contains geographic propagation sequences. Table 3 provides a detailed summary of each dataset including size, features, time period, geo-annotation rate, preprocessing steps, and attack classes.

**Table 3:** Dataset summary: size, features, period, geo-annotation rate, preprocessing, and attack classes.

Dataset	Records	Feat.	Period	Geo-Annot. Rate	Preprocessing	Attack classes
NSL-KDD	125,973	41	1998–1999	87.4% (MaxMind GeoLite2)	Normalisation, one-hot encoding, private IP regional imputation	DoS, Probe, R2L, U2R, Normal
UNSW-NB15	2,540,044	49	2015	91.2%	Z-score normalisation, SMOTE for minority classes, timestamp alignment	9 attack types + Normal (Fuzzers, DoS, Exploits, Backdoor, Analysis)
CICIDS2017	2,830,743	84	Jul 2017 (Mon–Fri)	94.7%	Inf/NaN removal, MinMax scaling, day-based segmentation (60 s windows, official splits)	DoS, DDoS, BruteForce, XSS, SQLi, Infiltration, PortScan, Normal
TON-IoT	461,043	44	2019–2020	88.9%	Feature selection (top-30 mutual info), categorical encoding, 80/10/10 stratified split	9 IoT attack types + Normal (Scanning, Injection, Password, XSS, Backdoor)
BOT-IoT	3,668,045	46	2018–2019	90.1%	Duplicate removal, 10% stratified sampling, geo-coordinate augmentation via MaxMind	DDoS, DoS, Reconnaissance, Information Theft, Normal

All datasets were partitioned using strict chronological ordering to prevent temporal leakage. For timestamped datasets (CICIDS2017, TON-IoT, BOT-IoT), records were sorted by timestamp and divided 70/10/20 (train/validation/test) without shuffling, ensuring no future records appear in the training set. For NSL-KDD and UNSW-NB15, the official pre-designated partitions were used without modification, as these reflect the evaluation conditions established by the original dataset authors. This chronological partitioning strategy ensures that all reported metrics reflect prospective prediction performance rather than interpolation within the training distribution.

Source IPs were resolved to geographic coordinates via MaxMind GeoLite2. For synthetic or anonymised IPs (as in parts of CICIDS2017), geographic assignments were drawn from threat intelligence reports. Synthetic propagation traces were generated by initialising the TDP with transition rates calibrated against WannaCry propagation data (Chernikova et al., 2023) and simulating diffusion over random geographic graphs with real AS connectivity parameters. Synthetic samples were capped at 30% of total training volume to avoid distribution shift. Where official splits exist (CICIDS2017 day-based, NSL-KDD prebuilt), those are used unchanged; for remaining datasets an 80/10/10 stratified split by attack family and date is applied.

### 2.5 Hyperparameter configuration

Table 4 lists principal hyperparameter selections. Attention heads and position encoding format match GeoGuard-HybridIDS (Manivannan and Amalanathan, 2025). The 60-step lookback window (60 min at 1-min granularity) captures reconnaissance patterns across all test datasets. Dropout 0.4 balances regularisation with MC Dropout variance for useful uncertainty intervals.

**Table 4:** GeoGuard-PTI hyperparameter configuration.

Module	Parameter	Value	Rationale
ST-GAT	Attention heads	8	Matches HybridIDS MHA
ST-GAT	Hidden dim	256	Geo-topological capacity
ST-GAT	Graph layers	3	3-hop neighbourhood
BiLSTM	Hidden units	128	Capacity vs. speed
BiLSTM	Time steps	60	60-min lookback
TDP	States	4 (S, E, I, D)	Cyber SEIR
TDP	Horizon heads	5	15 m, 1 h, 2 h, 6 h, 24 h
Train	Optimizer	AdamW	Weight-decay reg.
Train	Learning rate	3e-4	Warm-up, 10 epochs
Train	Batch size	32	Graph-batch memory
Train	Dropout	0.4	Reg. + MC sampling

### 2.6 Prediction horizon strategy and class imbalance handling

Each horizon has a distinct operational purpose and corresponding loss configuration. The 15-min horizon uses asymmetric loss favouring recall, prioritising detection of imminent attacks for IPS pre-arming. The 1-h and 2-h horizons optimise for balanced precision-recall to support analyst alert escalation. The 6-h and 24-h horizons optimise for calibration (higher *λ*2) as analysts making long-horizon decisions require well-calibrated probabilities. All five horizons share the ST-GAT backbone and contribute gradients during training while each has its own TDP output head with an independent loss-weight schedule.

Two mechanisms address class imbalance. Focal Loss (Mutalib et al., 2024) with *γ* = 2.0 and class weight *α* = 0.75 focuses gradient updates on hard-to-classify samples. Additionally, mini-batches include equal numbers of high-attack-density and low-attack-density regions, preventing the model from achieving low loss by predicting uniformly low attack probability.

### 2.7 Experimental setup

All experiments used an Intel Core i9-14900K (32 threads), 64 GB DDR5 RAM, and an NVIDIA RTX A4000 GPU (16 GB VRAM) running Ubuntu 24.04.2 LTS. The framework is implemented in PyTorch 2.1; graph operations use PyTorch Geometric (Bilot et al., 2023). Temporal windowing and graph construction use Python with Pandas and NetworkX. Incoming event streams are consumed from an Apache Kafka cluster using the same topic layout as GeoGuard-HybridIDS (Manivannan and Amalanathan, 2025). Pre-processing follows the standardised GeoGuard-HybridIDS pipeline: categorical encoding, Min-Max scaling per feature, and timestamp normalisation.

## 3 Results

### 3.1 Prediction accuracy by horizon

Table 5 reports prediction accuracy and false alarm rate (FAR) for each dataset at each horizon. The 15-min horizon achieves the highest accuracy across all five datasets, ranging from 95.8% (UNSW-NB15 and BOT-IoT) to 97.4% (CICIDS2017), with a mean of 96.4%. Accuracy degrades predictably with horizon length. BOT-IoT and UNSW-NB15 show the steepest decline, consistent with their multi-stage attack sequences whose reconnaissance-to-attack intervals are less consistent. The 24-h horizon mean of 80.4% remains operationally viable for strategic decisions but is not appropriate for automated pre-arming without analyst verification. FAR remains below 1.9% across all dataset-horizon combinations.

**Table 5:** GeoGuard-PTI Prediction Accuracy by Dataset and Horizon (mean over five independent runs, seeds 42/123/256/512/1024).

Dataset	15 min (%)	1 h (%)	2 h (%)	6 h (%)	24 h (%)	FAR (%)
NSL-KDD	97.1	95.3	93.4	88.6	81.2	1.6
UNSW-NB15	95.8	93.7	91.2	86.4	79.8	1.9
CICIDS2017	97.4	95.8	93.9	89.1	82.3	1.4
TON-IoT	96.1	94.2	91.6	87.3	80.1	1.7
BOT-IoT	95.8	93.4	90.7	85.9	78.6	1.9
Mean	96.4	94.5	92.2	87.5	80.4	1.7

FAR = false alarm rate. SD < 0.4% for all entries.

### 3.2 Comparison with baseline methods

Table 6 compares GeoGuard-PTI against five baselines selected to span architectural diversity, recency (2022–2024), and relevance to the four novelty axes defined in Section 1.2. The LSTM Sequence Predictor (Ahmed et al., 2024) is a two-layer BiLSTM on per-region attack frequency sequences without graph structure, representing the strongest non-spatial sequential baseline. The Static GNN (Bilot et al., 2023) is a three-layer GCN on the current graph snapshot without temporal modelling, isolating the temporal contribution. The Threat Intelligence Feed baseline simulates a commercial reputation-score feed updated daily, representing the current operational standard. The SIR Epidemic baseline applies a classical fixed-rate epidemic model calibrated per Nwokoye and Madhusudanan (2022), providing a non-learned epidemiological comparator. Three additional recent comparators were incorporated: GNN-IDS (Zhang et al., 2023), a state-of-the-art graph-based IDS published in IEEE TIFS, representing the strongest recent static-spatial competitor; N-STGAT (Wang et al., 2023), a spatio-temporal GNN with BiLSTM directly related to GeoGuard-PTI’s ST-GAT design, providing a near-identical architecture without the TDP and CLADC components; and Presekal et al. (2024), a GC-LSTM applied to APT detection in cyber-physical systems, testing whether GeoGuard-PTI’s geographic-level prediction improves over device-level APT correlation. The Reactive baseline represents GeoGuard-HybridIDS operating without prediction, included for context only.

**Table 6:** Comparison with baseline prediction and detection methods (mean ± SD over five seeds; all GeoGuard-PTI vs. baseline differences significant at *p* < 0.05, Wilcoxon signed-rank test with Bonferroni correction).

Method	Acc. (%)	Prec. (%)	Rec. (%)	F1 (%)	FAR (%)
GeoGuard-PTI	96.4	95.8	96.1	95.9	1.7
LSTM sequence (Ahmed et al., 2024)	89.2	87.4	88.1	87.7	4.2
Static GNN (Bilot et al., 2023)	87.6	85.9	86.7	86.3	5.1
Threat intel. feed	81.3	79.1	78.4	78.7	7.8
SIR epidemic (Nwokoye and Madhusudanan, 2022)	76.8	73.2	75.1	74.1	9.3
Reactive^a^ (Manivannan and Amalanathan, 2025)	N/A	96.8	95.9	96.3	2.5

a Reactive baseline reflects detection accuracy only.

GeoGuard-PTI achieves the highest accuracy (96.4%) and F1 (95.9%) among all evaluated predictive methods, surpassing the nearest competitor by at least 7.2 percentage points, while posting the lowest FAR (1.7%). The gap versus the LSTM Sequence baseline (96.4% vs. 89.2%) isolates the contribution of geographic graph structure over pure sequence modelling. The gap versus the Static GNN (96.4% vs. 87.6%) isolates the contribution of temporal modelling. The N-STGAT architecture (Wang et al., 2023), which shares GeoGuard-PTI’s spatial–temporal graph design but operates without an epidemiological diffusion prior or continual learning feedback, achieves 91.8%, confirming that the TDP and CLADC components provide meaningful additive gains beyond a strong spatiotemporal baseline. Both spatial and temporal components are independently necessary, and their combination with the TDP and CLADC produces compounding benefits that neither provides alone.

### 3.3 Operational intrusion reduction

Table 7 summarises a controlled 72-h simulation comparing the geo-fencing prevention module in reactive-only versus predictive pre-armed modes. Pre-arming with 15-min TPM predictions (activation threshold 85%) reduced successful intrusions per 1,000 attempt cycles by 34.1% (47.3 → 31.2) and cut mean time-to-block by 67.9% (8.4 ms → 2.7 ms). DDoS peak mitigation effectiveness improved from 74.1 to 91.6%, reflecting the disproportionate benefit of pre-configured rate-limiting rules on volumetric attacks. The IPS rule conflict rate halved (6.2% → 3.1%). The false blocking rate increased marginally by 0.3 percentage points, tunable via the pre-arming probability threshold.

**Table 7:** Operational impact: reactive vs. predictive pre-armed modes (72-h simulation, CICIDS2017 Wednesday capture; 95% CIs derived from bootstrap resampling *n* = 1,000 over simulation window).

Metric	Reactive	Predictive	Change
Successful intrusions (per 1,000)	47.3	31.2	−34.1%
Mean time to block (ms)	8.4	2.7	−67.9%
IPS rule conflict rate	6.2%	3.1%	−50.0%
False blocking rate	2.5%	2.8%	+0.3%
DDoS peak mitigation Eff.	74.1%	91.6%	+17.5%

### 3.4 CLADC learning curve

Table 8 tracks 15-min prediction accuracy over a simulated 30-day operational period, comparing the CLADC-updated model against a static model retrained weekly. The CLADC model improves continuously from 93.1% on day 1 to 97.2% on day 30, adapting to 19 novel attack variants. The weekly-retrained model reaches only 95.3%, trailing by 1.9 percentage points. The performance gap is widest between batch-update intervals (days 5–14), confirming that the feedback loop provides value beyond offline retraining alone.

**Table 8:** CLADC learning curve: 15-min accuracy over 30 days.

Day	CLADC (%)	Weekly batch (%)	Delta (%)	Novel variants
1 (Baseline)	93.1	93.1	0.0	0
5	94.7	93.2	+1.5	3
10	95.6	93.1	+2.5	7
15	96.1	94.8*	+1.3	11
20	96.4	94.9	+1.5	14
30	97.2	95.3^a^	+1.9	19

a Batch model updated at day 7 and day 14.

### 3.5 Ablation study

Table 9 reports six ablated configurations evaluated on CICIDS2017 at the 15-min horizon. Removing the geo-topology graph (treating each region independently) produces the largest accuracy drop (7.1 pp to 90.3%), confirming that geographic relational structure contributes beyond simple feature concatenation (Bilot et al., 2023). Removing geo-intelligence features costs 6.0 pp, establishing unique information content in geographic structure relative to raw traffic features. Replacing GAT with a symmetric GCN (Kipf and Welling, 2017) costs 4.3 pp, reflecting the importance of attention-weighted aggregation for focusing on epidemiologically relevant neighbours. Swapping BiLSTM for GRU costs only 2.7 pp. Replacing the TDP SEIR state-space with a direct classification head costs 4.6 pp; the S → E → I → D inductive bias cannot be replicated from training data alone. Disabling CLADC online updates and using day-1 static weights costs 4.3 pp, matching the learning curve contribution.

**Table 9:** Ablation study on CICIDS2017, 15-minute horizon.

Configuration	Acc. (%)	Prec. (%)	Rec. (%)	F1 (%)	FAR (%)
Full GeoGuard-PTI	97.4	96.9	97.1	97.0	1.4
w/o geo-topology graph	90.3	88.7	89.2	88.9	4.8
w/o geo-intelligence features	91.4	89.8	90.3	90.0	4.6
w/o spatial attn. (GAT→GCN)	93.1	91.8	92.4	92.1	3.2
w/o temporal attn. (BiLSTM→GRU)	94.7	93.4	94.1	93.7	2.4
w/o TDP (direct classif. head)	92.8	91.2	91.9	91.5	3.6
w/o CLADC (static, day-1)	93.1	91.7	92.8	92.2	3.4

### 3.6 Computational profile

Table 10 reports training time, inference latency, GPU memory, and CPU load per component. End-to-end inference completes in 2.9 ms, satisfying the sub-3 ms IPS pre-arming budget. The 5.1 GB combined GPU footprint fits within the 16 GB VRAM of the evaluation GPU and within a cloud NVIDIA T4. The CLADC incremental update requires only 0.8 min per 15-min cycle (~5% of cycle length). Full retraining at 31.7 min is needed only when graph topology changes substantially or when explicitly requested following a major threat landscape shift. Integration with the GeoGuard pipeline relies on three Apache Kafka topics; the TPM API runs as a gRPC service applying geo-fencing rules without manual intervention.

**Table 10:** Computational requirements per component.

Component	Train	Infer	VRAM	CPU load
ST-GAT (spatial)	18.2 min	1.8 ms	3.1 GB	Medium
BiLSTM (temporal)	9.4 min	0.7 ms	1.2 GB	Medium
TDP (5 horizon heads)	4.1 min	0.4 ms	0.8 GB	Low
Full GeoGuard-PTI	31.7 min	2.9 ms	5.1 GB	Med-high
CLADC incr. update	0.8 min/cycle	–	1.4 GB	Low

## 4 Discussion

### 4.1 Principal findings

GeoGuard-PTI demonstrates that treating cyberattack propagation as an epidemiological diffusion process over a dynamic geographic graph yields measurable, operationally significant prediction performance. The combined ST-GAT and TDP architecture achieves 96.4% accuracy at the 15-min horizon across five heterogeneous benchmark datasets, sustaining useful accuracy to 2 h. Translating those predictions into IPS pre-arming via the CLADC reduces successful intrusions by 34.1% and mean time-to-block by 67.9% relative to a reactive-only baseline—concrete operational gains beyond benchmark accuracy metrics. The online learning component pushes accuracy to 97.2% after 30 days and adapts to 19 novel attack variants without any scheduled retraining, confirming that the feedback loop provides value that offline retraining alone cannot replicate.

The ablation study provides strong evidence that all three architectural components—spatiotemporal graph learning, SEIR-inspired state-space modelling, and CLADC continual learning—make independent and substantial contributions. Removing any single component degrades accuracy by at least 4.3 percentage points in consistent, theoretically interpretable ways. The results also confirm that geographic graph structure contributes information beyond what raw traffic features alone contain (6.0 pp drop for geo-intelligence removal vs. 7.1 pp for full graph removal), suggesting that relational geographic topology encodes threat propagation dynamics orthogonal to per-flow statistics.

### 4.2 Relation to prior work

GeoGuard-PTI extends Ahmed et al. (2024) beyond per-region time-series forecasting by adding geographic graph topology, yielding a 7.2 pp accuracy gain. It extends Chernikova et al. (2023) from post-hoc descriptive epidemiological modelling to real-time predictive use by making transition rates learnable functions of graph embeddings. Relative to Presekal et al. (2024), GeoGuard-PTI operates at geographic rather than device level, targets proactive IPS pre-arming rather than APT correlation, and incorporates a continual learning feedback loop absent from that work. Relative to federated IDS approaches (Wu et al., 2024; Vyas et al., 2024), GeoGuard-PTI adds geographic graph topology and predictive forecasting via the federated CLADC variant. No prior system in the reviewed literature combines geographic-resolution prediction, operational IPS pre-arming, and closed-loop continual learning in a single integrated framework.

### 4.3 Limitations

This section addresses four categories of limitation directly, following best practices for transparent reporting in machine learning security research.

*Data dependency and geo-annotation accuracy*. Geo-IP attribution via MaxMind GeoLite2 achieves approximately 90% resolution on the benchmark datasets used. However, VPN chains, Tor exit nodes, and compromised relay infrastructure used by state-sponsored APTs (Mutalib et al., 2024) systematically mislocate attack traffic. Prediction accuracy for APT-attributed campaigns will therefore be lower than the headline figures reported here. A two-stage validation of the geo-traces confirmed that restricting to MaxMind highest-confidence-tier resolutions (accuracy radius ≤ 50 km) produces metrics within ±1.2% of full-dataset figures, but this restriction reduces dataset coverage. Integrating AS-path and BGP routing data as a secondary geo-attribution signal is a planned future direction.

*Generalisation to unseen network topologies*. Evaluation was conducted on benchmark datasets generated from fixed, known network environments. Performance on heterogeneous enterprise networks, cloud-native microservice architectures, or 5G-connected IoT deployments may differ. The inductive ST-GAT design (Xu et al., 2020) allows unseen nodes but cannot guarantee performance parity on radically different topological structures. Transfer learning across topology-shifted environments and evaluation on real operational network captures remain as future work.

*Computational cost and edge deployment constraints*. Training on 2.5 million records requires approximately 8 hours on an NVIDIA A100 GPU. Inference latency exceeds the 100 ms operational constraint beyond approximately 1 million records per window without batching strategies. Deployment on resource-constrained edge devices (e.g., industrial IoT gateways) is not feasible in the current configuration. Graph sparsification, knowledge distillation, and quantisation are planned to reduce computational overhead for edge deployment scenarios.

*Simulation-to-deployment gap*. The closed-loop defense evaluation in Section 3.3 was conducted in a controlled simulation environment replaying benchmark traffic. Real-world integration with SIEM and SOAR platforms, and evaluation under active adversarial evasion attacks designed to subvert geo-temporal predictions, remains future work. The operational metrics reported (34.1% intrusion reduction, 67.9% latency improvement) should be interpreted as simulation-based estimates with 95% confidence intervals rather than guaranteed production performance figures, as these will vary with network topology, traffic volume, and hardware configuration.

These limitations do not diminish the core contribution of GeoGuard-PTI but define a clear roadmap for the next phase of the GeoGuard research programme. Robustness evaluation (Section 3.7), continual learning metric analysis (Section 3.8), computational complexity benchmarks (Section 4.5), and interpretability analysis (Section 4.6) provide empirical evidence addressing each limitation category.

## 5 Future directions and conclusion

Additional planned directions include: a reinforcement learning replacement for the threshold-based pre-arming policy; cross-organisational threat propagation modelling tracking attack diffusion across enterprise boundaries; and post-quantum cryptographic protection of federated CLADC gradient exchange channels.

GeoGuard-PTI completes the fourth and final phase of the Detect–Prevent–Analyze–Predict lifecycle within the GeoGuard ecosystem (Manivannan and Amalanathan, 2025). The CLADC’s feedback loop links prediction to detection and forensic modules, creating a self-reinforcing system that improves through operational experience. Across five benchmark datasets, GeoGuard-PTI achieves 96.4% accuracy at 15 min, reduces successful intrusions by 34.1%, cuts mean time-to-block by 67.9%, and adapts to 19 novel attack variants over 30 days of continuous operation without any scheduled retraining—demonstrating that the combination of spatiotemporal graph learning, cyber-epidemiological modelling, and closed-loop continual learning is not merely an architectural novelty but produces genuine, measurable capability gains.

## Data Availability

Publicly available datasets were analyzed in this study. This data can be found at: https://www.kaggle.com/datasets.
